# From engineering to editing the rat genome

**DOI:** 10.1007/s00335-017-9705-8

**Published:** 2017-07-27

**Authors:** Stephen Meek, Tomoji Mashimo, Tom Burdon

**Affiliations:** 1 The Roslin Institute and R(D)VS, University of Edinburgh, Easter Bush, Midlothian, EH25 9RG UK; 2 Institute of Experimental Animal Sciences, Graduate School of Medicine, Osaka University, Osaka, 565-0871 Japan; 3 Genome Editing Research and Development (R&D) Center, Graduate School of Medicine, Osaka University, Osaka, 565-0871 Japan

## Abstract

Since its domestication over 100 years ago, the laboratory rat has been the preferred experimental animal in many areas of biomedical research (Lindsey and Baker The laboratory rat. Academic, New York, pp 1–52, 2006). Its physiology, size, genetics, reproductive cycle, cognitive and behavioural characteristics have made it a particularly useful animal model for studying many human disorders and diseases. Indeed, through selective breeding programmes numerous strains have been derived that are now the mainstay of research on hypertension, obesity and neurobiology (Okamoto and Aoki Jpn Circ J 27:282–293, 1963; Zucker and Zucker J Hered 52(6):275–278, 1961). Despite this wealth of genetic and phenotypic diversity, the ability to manipulate and interrogate the genetic basis of existing phenotypes in rat strains and the methodology to generate new rat models has lagged significantly behind the advances made with its close cousin, the laboratory mouse. However, recent technical developments in stem cell biology and genetic engineering have again brought the rat to the forefront of biomedical studies and enabled researchers to exploit the increasingly accessible wealth of genome sequence information. In this review, we will describe how a breakthrough in understanding the molecular basis of self-renewal of the pluripotent founder cells of the mammalian embryo, embryonic stem (ES) cells, enabled the derivation of rat ES cells and their application in transgenesis. We will also describe the remarkable progress that has been made in the development of gene editing enzymes that enable the generation of transgenic rats directly through targeted genetic modifications in the genomes of zygotes. The simplicity, efficiency and cost-effectiveness of the CRISPR/Cas gene editing system, in particular, mean that the ability to engineer the rat genome is no longer a limiting factor. The selection of suitable targets and gene modifications will now become a priority: a challenge where ES culture and gene editing technologies can play complementary roles in generating accurate bespoke rat models for studying biological processes and modelling human disease.

## Introduction

The study of human development and disease requires the use of animal models. No single model system is likely to be able to accurately mimic all human phenotypes, or alone lead to the discovery of drugs with the desired efficacy in humans. The choice of the most appropriate model system is dependent on the biological question whilst taking into account experimental considerations and the ability to reliably report directly on the human disease. Cost-effective animal husbandry has resulted in a rodent model being the preferred option for many studies. For many reasons the rat has been preferred over the mouse for studies of physiology, neurobiology, pharmacology and behaviour. Despite both being members of the same family, and sharing many common features, rat and mouse are distinctly different species, evolutionarily separated by 15–20 million years—a significant degree of separation that has resulted in a number of functional and behavioural differences (Makalowski and Boguski 1998). Evolved differences in social and foraging behaviours and stress-coping strategies have meant that the rat is better suited to many behavioural tasks, and well-established behavioural tests have been developed and validated for the rat. Adapting some of these tests to the mouse has often been impossible or difficult due to differences in behaviour (Parker et al. 2014). The rat is less stressed by human handling than the mouse, which minimises any influence or variability on experimental test results. These behavioural differences also impact on other aspects of experimental investigation. The lower stress levels in the rat aids live animal imaging techniques by avoiding the requirement for anaesthetics that can interfere with normal brain activity (Febo 2011; Harris et al. 2015), and the larger brain of the rat affords better image resolution. Indeed, the larger size of the rat facilitates experimental procedures that were difficult or impossible to perform in the mouse such as the introduction of a blood vessel catheter (Feduccia and Duvauchelle 2010) or procedures that cause too much damage in the smaller brain of the mouse such as introduction of an intracerebral cannula (Kokare et al. 2011). There are also functional differences that make the rat more relevant for studying human disease. For example, unlike the mouse, in rats and humans the serotonin receptor subtype 5-HT6, which is implicated in a variety of mood disorders, is enriched in the basal ganglia (Hirst et al. 2003). The pharmacological profile of the mouse receptor also differs from that of rat and human accounting for differences in ligand binding affinity. The rat is the major model system for the pharmaceutical industry because of similarities with humans in drug binding affinities and toxicological profiles, and almost every new drug is tested in the rat at some point.

Despite the many advantages of the rat as an experimental model, the mouse was always the preferred choice in the field of genetics. This was initially due to its smaller size and shorter reproductive cycle, but this preference was cemented following the derivation of mouse embryonic stem cells (ES) in 1981 (Evans and Kaufman 1981; Martin 1981). ES cells have facilitated the application of genome engineering technologies to create precise genetic modifications within the mouse genome in vitro and have provided a route to the generation of custom-made mouse models of human disease (Capecchi 2005). However, equivalent rat ES cell lines have not been available until relatively recently (Buehr et al. 2008; Li et al. 2008). Whilst, the development of mouse ES cells has led to the production of numerous invaluable genetically modified mouse lines for biomedical research, these technological advantages are restricted by the limits of the mouse model system, the ability to record reliable data and the accuracy with which it reflects the human disease. For example, mouse models of Huntington’s disease have shown either no symptoms or only a rapid-onset phenotype with limited usefulness for neurotransplantation and in vivo imaging. In contrast, the transgenic rat model exhibited adult-onset phenotypes, which included a slowly progressive motor dysfunction and histopathological and metabolic alterations typical of Huntington’s (von Horsten et al. 2003).

The recognised value of the rat as a biomedical model brought about a concerted effort by a committed and diverse research community, to establish the necessary tools and resources to assist in developing and exploiting new genome editing technologies and enabling comparative genomics (Table 1). These efforts were supported by major commitments from European Union and NIH–NHLBI-funded projects to develop alternative approaches to circumvent the absence of bonafide rat ES cells. Some of the challenges in establishing reliable methods for collecting and manipulating rat embryos arise from idiosyncrasies of rat reproduction and preimplantation development. For example, the induction of ovulation through injection of pregnant mare gonadotrophin, although effective in mice, is unreliable and inefficient in rats. This difficulty was overcome by delivering partially purified, pig pancreas derived-follicle stimulating hormone using osmotic minipumps, which markedly increases the yield of ova (Armstrong and Opavsky 1988; Charreau et al. 1996). Furthermore, culture conditions had to be established to limit the spontaneous activation of rat oocytes (Keefer and Schuetz 1982), and reliably support preimplantation development, thus maintaining the biological potential of rat embryos in culture (Miyoshi 2016). Indeed, pronuclear microinjection has been used to generate transgenic rats since the early 1990s, mainly for gain-of-function studies (Mullins et al. 1990; von Horsten et al. 2003; Popova et al. 2005; Leon et al. 2010). Phenotype-driven, forward genetic approaches such as chemically induced *N*-ethyl-*N*-nitrosourea (ENU) mutagenesis (Zan et al. 2003; Smits et al. 2004) and transposon-based gene-trap systems (Lu et al. 2007; Kitada et al. 2009; Ivics et al. 2014; Li et al. 2016) have been very successful at randomly generating knock-out rats. The derivation and culture of spermatogonial stem cells (Hamra et al. 2005; Wu et al. 2009) together with robust screening methods (Yanagihara and Mizuuchi 2002; Mashimo et al. 2008; Izsvák et al. 2010) have improved the efficiency of these approaches by reducing the ethical and financial cost, allowing in vitro screening, cryogenic preservation and generation of transgenic rats via intra-cytoplasmic sperm injection (ICSI) (Dozortsev et al. 1998). The detailed benefits and limitations of these approaches have been thoroughly reviewed elsewhere (Tesson et al. 2005; Jacob et al. 2010). In this review, we will discuss recent technological advances made in the field of reverse genetics and site-specific gene editing, namely the derivation of rat ES cells and the utilisation of the CRISPR/Cas site-specific nuclease system. Application of these powerful genomic tools has now overcome the major barriers to applying genetic modification to the rat, and allows researchers to tap into the wealth of established physiological and behavioural data already available for this useful laboratory rodent. This will permit genetic dissection of established natural disease rat models and accelerate the generation of new and better genetic and phenotypic rat models of human disease.

**Table 1:** List of rat resources

Resource	Available resources	Reference
Rat Genome Database	Genetic, genomic, phenotype and disease data consisting of strain, gene and QTL reports, mapping data, microsatellite markers, sequence data and software tools http://www.rgd.mcw.edu/	Twigger et al. (2002)
PhysGen	Phenotype database of 45 FHH and SS consomic strains http://www.pga.mcw.edu/	Wang et al. (2015b)
MCW Gene Editing Rat Resource Centre	Funded to generate ~200 knock-out rat strains based on nominated genes involved in hypertension and renal disease http://www.rgd.mcw.edu/wg/gerrc	
National Bioresource Project for the Rat (NBPR)	Repository of >700 rat strains and sub-strains including reporter, Cre and disease lines, cryopreserved embryos and sperm http://www.anim.med.kyoto-u.ac.jp/nbr/repository.aspx	Mashimo et al. (2005, 2008), Serikawa et al. (2009)
	The Rat Phenome Project hosts phenotypic data for 109 parameters collected from >200 inbred rat strains http://www.anim.med.kyoto-u.ac.jp/nbr/phenome.aspx	
	BAC library (BAC end sequencing of F344/Stm and LE/Stm) http://www.anim.med.kyoto-u.ac.jp/nbr/gbrowser.aspx http://www.dna.brc.riken.jp/en/NBRPraten.html	
	Kyoto University rat ENU mutant archive (KURMA). >5000 G1 DNA and sperm samples http://www.anim.med.kyoto-u.ac.jp/enu/home.aspx	
Rat Resource and Research Centre (RRRC)	Repository of >350 rat strains and sub-strains including reporter, Cre and disease lines, cryopreserved embryos, sperm and ES cells http://www.rrrc.us/	

## Embryonic stem cells

Embryonic stem (ES) cell lines are derived from pluripotent cells within the inner cell mass of the blastocyst, and are defined by three cardinal properties: (1) they undergo unlimited self-renewal and are effectively immortal, (2) they are pluripotent and differentiate into all foetal cell types including the germ cells and (3) they can engraft into a host embryo and resume normal development to generate chimaeric animals. The combination of these three properties makes ES cells a powerful system with which to explore gene function in vitro and in vivo. Indeed, the successful derivation of mouse ES cells was a major advance in the development of targeted genome engineering technologies in the mouse using homologous recombination and the generation of genetically modified mouse models of human disease (Evans and Kaufman 1981; Martin 1981; Capecchi 2005). Nevertheless, the limitations of the mouse as an experimental model also fueled efforts to derive equivalent stem cells from species better suited to particular areas of biomedical research. Unfortunately the standard mouse ES cell culture conditions, consisting of serum and the cytokine LIF (leukaemia inhibitory factor), does not support the derivation and expansion of bona fide germline competent ES cells from the blastocysts of most other species, including the rat. Under these conditions, the stem cell compartment of the rat blastocyst usually differentiates to form extraembryonic cell types (Buehr et al. 2003), consistent with LIF supporting the growth of rat yolk sac precursors (Chuykin et al. 2010; Debeb et al. 2009), thereby undermining the establishment of undifferentiated pluripotent rat cell lines. The crucial breakthrough in deriving genuine rat ES cells, however, arose from ground-breaking studies in mouse ES cells that identified key signalling pathways that control self-renewal and differentiation (Burdon et al. 1999; Chen et al. 2006; Kunath et al. 2007; Stavridis et al. 2007; Ying et al. 2008; Wray et al. 2011; Yi et al. 2011). These studies showed that whilst the ES cell regulatory network supports stem cell self-renewal it also simultaneously poises the cells ready to differentiate, leading to the suggestion that uncoupling the effects of these intrinsic differentiation signals was likely to be the key to successful ES cell derivation.

These pioneering studies highlighted two important signalling pathways that promote mouse ES cell differentiation. The activation of MAPK (mitogen-activated protein kinase) through auto-inductive FGF4 (fibroblast growth factor 4) signalling destabilises pluripotency (Kunath et al. 2007; Stavridis et al. 2007). However, suppression of this pathway by blocking FGF receptor activity or by more direct disruption of MAPK signalling using a small molecule inhibitor of the MAPK activator MEK, uncouples this differentiation signal and shifts the balance in favour of ES cell self-renewal (Burdon et al. 1999; Chen et al. 2006; Ying et al. 2008). The other key differentiation signal is the transcriptional repressor TCF3/TCF7L1, which suppresses expression of a number of essential ES cell regulators (Sokol 2011). Wnt signalling or inhibition of its downstream target glycogen synthase kinase 3 (GSK3) induces β-catenin activity (Ding et al. 2000; ten Berge et al. 2011) which in turn destabilises TCF3/TCF7L1 and thereby promotes ES cell self-renewal (Wray et al. 2011; Yi et al. 2011). By combining inhibitors of FGFR, MEK and GSK3 signalling in a serum-free medium (3i:FGFR + MEK + GSK3 inhibitor or 2i:MEK + GSK3 inhibitor culture), researchers demonstrated that ES cells could be derived with high efficiency, not only from mouse strains previously regarded as non-permissive for ES cell derivation (Ying et al. 2008; Nichols et al. 2009), but also most importantly from the rat (Buehr et al. 2008; Li et al. 2008).

## Rat ES cell derivation

The first rat ES cell studies described the derivation of cell lines from DA, Sprague Dawley (SD) and Fisher F344 strains (Buehr et al. 2008; Li et al. 2008), with subsequent reports extending this to include Lewis (Meek et al. 2013), Brown Norway (Zhao et al. 2010), Wistar and Long Evans (Kawamata and Ochiya 2010). Collectively these reports suggested that the rat genetic background is not a major barrier to establishing cell lines. Furthermore, in contrast to that reported for mouse ES cell lines cultured in serum/LIF (Schwartzberg et al. 1989), there is no robust evidence demonstrating strain incompatibility between rat ES cell and the host embryo for chimaera generation (Buehr et al. 2008; Hirabayashi et al. 2010; Blair et al. 2012; Hong et al. 2012; Meek et al. 2013). Nevertheless, the culture conditions and the genetic background of rat strains may influence the long-term stability of the phenotype and karyotype of rat ES cells in culture, and contribute to the variability seen in germ line transmission obtained using rat ES cells (Buehr et al. 2008; Tong et al. 2010; Blair et al. 2012).

In one of the first studies it was shown that supplementation of 2i medium with LIF improved rat ES cell growth (Buehr et al. 2008), a finding supported by the observation that overexpression of the key LIF target, STAT3, restricted the differentiation of rat ES cells (Li et al. 2008). Careful titration of the inhibitors used in the 2i medium can also affect rat ES cell self-renewal. In particular, the dose of GSK3 inhibitor (GSK3i), which determines the level of stable β-catenin, plays a role in modulating the balance between self-renewal and differentiation. Unlike mouse, rat ES cells are rather sensitive to the level of GSK3 inhibition, largely because they express elevated levels of the β-catenin regulated transcription factor LEF1, which triggers the activation of differentiation genes. As a consequence, an appropriate level of GSK3i is required to avoid destabilising rat ES cell self-renewal (Chen et al. 2013; Meek et al. 2013). In a more radical modification of the standard inhibitor formulation, supplementation of 2i medium with inhibitors of Rho-associated kinase (Rock inhibitor Y-27632) and a transforming growth factor-β inhibitor (TGFβi: A-83-01), combined with 20% foetal bovine serum, was reported to support efficient derivation of germline competent ES cells from three different strains of rat (Kawamata and Ochiya 2010). Although it is not clear what specific benefits are afforded by the addition of serum and TGFβ inhibition, the Rock inhibitor is widely used to protect human ES cells and epiblast stem cells from apoptosis induced by disaggregation at passaging (Watanabe et al. 2007; Ohgushi et al. 2010)—and may prove to have a similar supportive function in rat ES cell cultures. It has also been reported that inhibition of protein kinase C (PKC) alone can maintain cell lines previously established in 2i, and for specific strains may even be sufficient to derive cell lines de novo (Rajendran et al. 2013). Significantly, it has been shown recently that PKC inhibition in combination with 2i medium allows the isolation of human blastocyst derived cell lines that appear to be analogous to rodent ES cells (Guo et al. 2016). Whilst further culture optimization may be required to ensure stable and robust expansion of rat ES cells in culture, it is clear that the 2i + LIF condition has been sufficient for the derivation of germline competent rat ES cells in many laboratories, and has paved the way for exploitation of genome engineering technologies in this valued species.

## Gene targeting in rat ES cells

The first reports describing the genetic modification of rat ES cells and their use in generating transgenic rats appeared in 2010. Hirayabashi et al. demonstrated that Brown Norway rat ES cells could be stably transfected with a fluorescent Kusabira-Orange reporter gene using electroporation, and could engraft host embryos and be successfully passed through the germ line (Hirabayashi et al. 2010). Similarly, ES cell-derived transgenic rats, carrying an Oct4-Venus stem cell-specific transgene, were generated that allowed tracking of Oct4 expression in transgenic blastocyst outgrowths and in the embryonic testis (Kawamata and Ochiya 2010). The first demonstration of germline transmission of targeted rat ES cells was reported in the same year, where Tong and colleagues generated a mutant rat lacking the p53 tumour suppressor gene (Tong et al. 2010). The mutant rats were prone to early development of cancer, consistent with previously described mouse p53 mutants, but interestingly the spectrum of tumour types was somewhat different in the two species (Yan et al. 2012). The predominant tumour type in the mutant rats was early onset spontaneous hemangiosarcomas, whereas in mice the most frequent tumours were lymphomas. Notably, an ENU-induced p53 mutant rat reported in another study also displayed a bias for sarcomas, supporting the preferential development of this tumour type in the rat (van Boxtel et al. 2010). The p53 heterozygous rats also displayed a delay in tumour onset and a wider spectrum of tumour types, including breast cancer which suggested that these mutant rats might prove to be a useful model for the p53-associated Li-Fraumeni syndrome in humans.

In the wake of the p53 knock-out study, a number of other laboratories have reported the use of homologous recombination to generate new lines of ES cell-derived mutant rats. Yamamoto and colleagues disrupted the protease-activated receptor 2 (PAR-2) gene, a member of a family of G-protein-coupled receptors that regulate smooth muscle activity, modulate inflammatory responses, and are potential therapeutic targets in some human diseases (Cocks et al. 1999; Kawagoe et al. 2002; Kawabata 2003; Yamamoto et al. 2012). Meek and co-workers inactivated the gene encoding hypoxanthine phosphoribosyltransferase (HPRT), a key regulator of the purine salvage pathway, generating a mutation that in humans causes the debilitating neurological disease Lesch–Nyhan Syndrome (Lesch and Nyhan 1964). The *Hprt* mutant rats showed a deficit in dopamine in their brains, consistent with observations in human Lesch–Nyhan patients and mutant mice, and may therefore represent a suitable animal model to investigate the behavioural changes associated with HPRT deficiency (Meek et al. 2016). In a further demonstration of the utility of the rat model, Uenoyama and colleagues knocked out the gene encoding rat Kisspeptin, a neuropeptide involved in the regulation of puberty and reproduction (Irwig et al. 2004; Matsui et al. 2004). The larger size of the rat allowed a detailed analysis of hormonal profiles, exposing disruption of normal pulsatile and surge patterns of gonadotropin secretion in Kisspeptin deficient rats (Uenoyama et al. 2015).

In addition to these standard gene knock-out experiments, more sophisticated targeting protocols routinely used in mouse have also been applied to rat ES cells. This includes the targeted “knock-in” of transgenes as demonstrated by replacement of the rat Kynurenine aminotransferase II gene with a human cDNA, and the insertion of a ubiquitous and nuclear-targeted histone 2B-tdTomato fluorescent reporter transgene into the ROSA26 locus (Yamamoto et al. 2015; Kobayashi et al. 2012). Consistent with mouse studies, the ROSA26 locus provided a “safe harbour” for transgene insertion and a constitutive pattern of transgene expression in vivo (Goto et al. 2015; Kobayashi et al. 2012). Injection of the tdTomato-reporter cells into mouse blastocysts generated interspecies chimaeras, thus demonstrating the utility of the rat cells in blastocyst complementation experiments and their capacity to generate organs in a heterologous host.

The inclusion of a drug-resistance expression cassette in targeting vectors is usually required to facilitate selection of transfected ES cell clones, but can have adverse effects on regulation of a host target gene. This is usually solved by recombinase-mediated excision of the selective cassette in a second round of clonal selection. Unfortunately, this additional step increases the chances of rat ES cell clones acquiring karyotypic anomalies and the loss of developmental potential. However, by using a clever technical trick, this second round of cloning can be avoided by incorporating into the cassette a transgene that drives testes-specific expression of a recombinase, thus generating a self-excising cassette that deletes itself when the ES cells pass through the male germ line. This useful technical modification obviates the requirement for a second round of clonal selection, and should help to improve the retention of germ line potential in targeted rat ES cells (Lan et al. 2016).

Whilst some concerns may remain over the stability of rat ES cells in culture, it is clear from these recent successes that rat ES cell lines are useful for generating genetically modified rats. Available data indicate that rat ES cell derivation is robust and efficient, and the reported frequency of homologous recombination in rat ES cells is equivalent to those typically seen in mouse ES cells. The contribution of rat ES cells to the germline of chimaeric rats does vary between studies, but the high efficiencies achieved in some cases suggest that in under the right conditions rat ES cells can provide a highly effective route for introducing targeted mutations into the rat.

## Gene editing in the rat

**Fig. 1:**
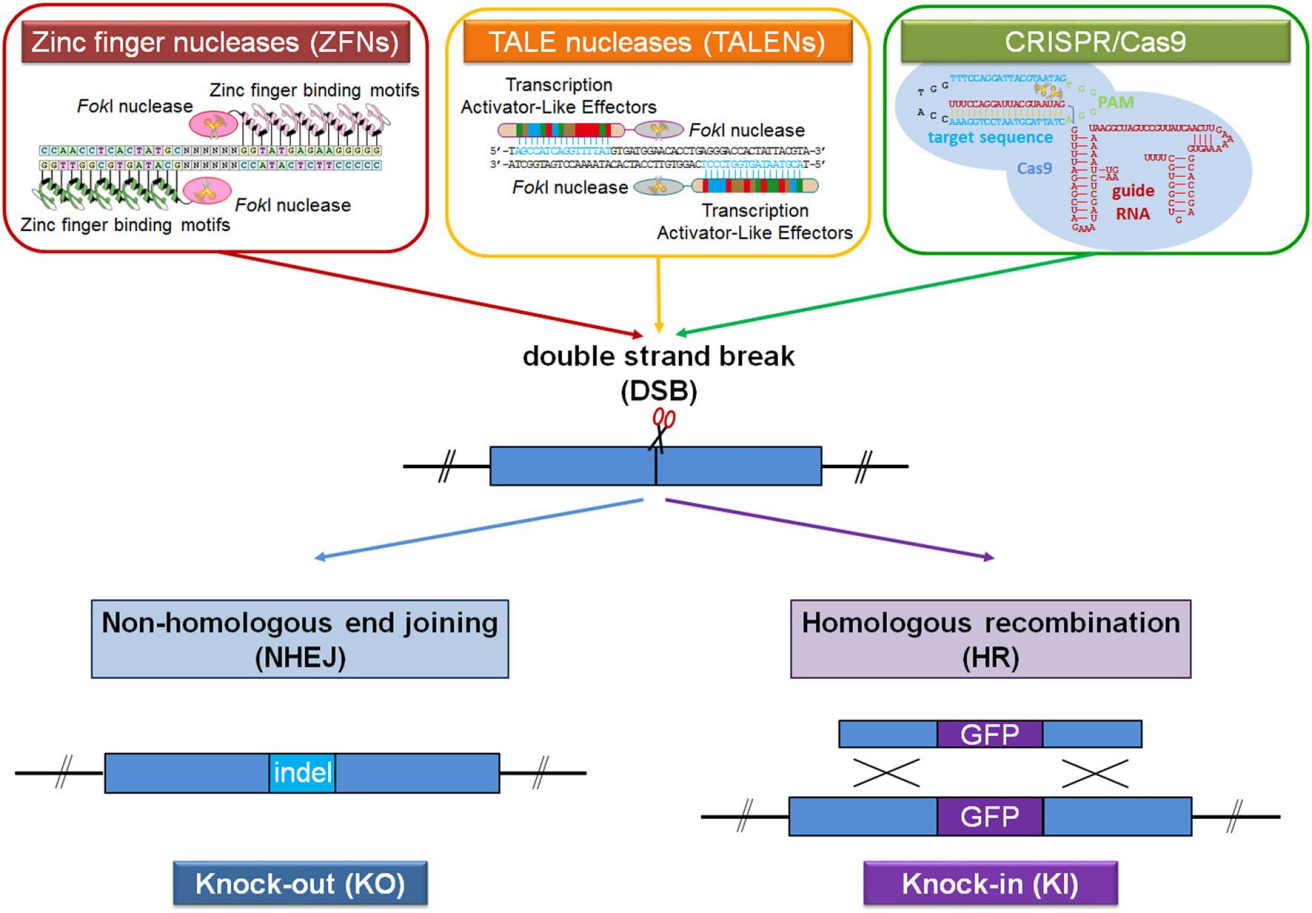
Summary of site-specific nuclease gene editing tools. Site-specific nucleases consist of a DNA-specific binding domain fused to a nuclease domain. In the case of ZFNs and TALENs, the nuclease domain is derived from the *Fok*I restriction endonuclease. The formation of *Fok*I homodimers is required for cleavage to occur. This is achieved by using pairs of ZFNs and TALENs designed to opposite strands and flanking the cut site. CRISPR-mediated cleavage is achieved using the RNA-guided DNA nuclease, Cas9. Following the generation of a DSB, the DNA can be repaired either by NHEJ, which can generate knock-out mutations resulting from the introduction of random insertions or deletions (indels), or more precisely by homology-directed repair using a homologous DNA template containing the desired modification to be inserted

The development of synthetic sequence-specific nucleases, commonly termed gene editors, has transformed prospects for introducing germ line modifications in laboratory animals and in livestock. Indeed, for researchers working with the rat, the enzymes represent a tool that elevates genetic modification in the rat to parity with the methods available for the mouse. The injection of gene editor mRNA or protein directly into zygotes is a highly effective way of introducing targeted mutations into the rat germ line and in many instances can substitute for manipulations normally performed in embryonic stem cells. The three types of gene editors, zinc finger nucleases (ZFN), TALENs and CRISPR/Cas systems have all been used to genetically modify the rat genome, and the type of mutations they generate can be broadly organised into two main categories (Fig. 1) (Geurts et al. 2009; Tesson et al. 2011; Li et al. 2013a, b). In the first category, the introduction of a double-stranded break into a target gene by an editor enzyme induces the non-homologous end joining (NHEJ) DNA repair mechanism that re-ligates the exposed ends. If repaired correctly, the target site can serve as a substrate for another round of cleavage by an editor enzyme. If however, the exposed ends of DNA are subject to nuclease attack and then re-ligated, this leads to deletions of sequences ranging from a few base pairs to hundreds of base pairs, some of which may generate a functionally null allele. Extraneous DNA sequences can also be inserted at the cleavage site, although this occurs less frequently than deletion. A major advantage of NHEJ mode of mutagenesis is that it is simple and effective, relying only on delivery of the gene editor and the imprecision of this DNA repair mechanism. However, a major disadvantage is that the researcher has little control over the type of mutation that is generated, even though in some instances short regions of (micro) homology between the cleaved ends of a chromosome can bias the outcome. In the second category, the inclusion of a DNA template molecule with homology to sequences on either side of the cleavage site is used by a homology-dependent repair (HDR) mechanism to mend the damaged chromosome. This process can be highly efficient, but its main advantage is that it allows the introduction of precise template-directed modifications at a target site, including the introduction of novel DNA sequences.

CRISPR/Cas is now generally considered the preferred gene editing system, due to its flexibility, ease of use and low cost. However, genetically modified rats have been generated using the other platforms, and represent a useful resource for researchers using the rat as their chosen model system. In this section, we will therefore briefly mention how the three most popular approaches to gene editing in rats evolved, starting with ZFN, moving to TALENs and then concluding with a description of the contemporary CRISPR/Cas-based systems. Meganucleases, a fourth editing platform utilising engineered forms of naturally occurring restriction enzymes derived from lower order animals and plants, have also been used to introduce targeted mutations into the rat genome (Ménoret et al. 2013) but has not been widely adopted and will not be discussed further here.

## Editing using zinc finger nucleases

ZFNs are synthetic restriction enzymes that carry an array of engineered sequence-specific zinc finger DNA binding units linked to the nuclease domain of the restriction enzyme Fok1. The adjacent binding of two properly orientated and spaced ZFNs on DNA promotes dimerisation of the Fok1 domain which then cleaves the DNA within the spacer region between the ZFN binding sites (Porteus and Carroll 2005). Although ZFNs can be designed to cleave many sequences across the genome, the sequence specificity of the zinc finger domains combined with the complexity of the interactions between domains mean that producing a ZFN is a complicated and non-trivial task, outwith the routine scope of many laboratories. Nevertheless, ZFNs were used in the first report of gene editing in rats, which showed that these synthetic nucleases were highly effective in introducing NHEJ mutations in different target genes at frequencies ranging from 5 to 75% of live pups born (Geurts et al. 2009). The high efficiencies and short time frames achieved in this first study propelled ZFNs to the forefront of rat transgenesis and the laboratories of Geurts, Dwinell and Jacob embarked on a large-scale programme to knock-out ~200 rat genes associated with hypertension and renal disease as part of an NIH-funded resource programme (http://rgd.mcw.edu/wg/gerrc). A number of reports of disease-associated mutations in specific target genes have arisen from this programme, including work in which six genes in a locus identified in a genome wide association study (GWAS) were mutated individually to assess their contribution to a renal phenotype (Flister et al. 2013). Other laboratories also embraced the ZFN technology to generate immunodeficient (SCID) rats that are suitable recipients for transplantation experiments, carrying mutations in the IL2-receptor gamma and *Prkdc* genes (Mashimo et al. 2010, 2012). Cui and colleagues also demonstrated the feasibility using ZFNs to direct homology-dependent repair by successfully introducing GFP reporters into the *Mdr1a* and *PXR* genes of Sprague Dawley and Long Evans rats (Cui et al. 2011).

Although constitutive disruption of a target gene’s function can be highly informative, there are many instances where conditional ablation of a gene product in a tissue-specific or temporally dependent manner is required. The most common way to achieve this is to create an allele where an essential exonic region is flanked with LoxP recombination sites, that when induced to recombine through expression of CRE recombinase delete the essential region and inactivate the target gene. Brown and colleagues used two pairs of ZFNs and plasmid-mediated HDR in a two-cut strategy to insert LoxP sites in intronic DNA surrounding exon 4 of the gene encoding the NMDA receptor subunit *Grin* (Brown et al. 2013). By crossing rats carrying the floxed Grin allele with a transgenic line of rats expressing CRE recombinase under the control of the endogenous tyrosine hydroxylase (*Th*) promoter, selective deletion of Grin function was obtained in Th—expressing brain and adrenal gland tissue, demonstrating the utility of this ZFN-mediated approach to generate conditional alleles in the rat. In summary, although largely superseded now by more flexible and less costly alternatives, ZFNs have proved to be highly effective and relatively specific, with low levels of off-target effects being reported.

## TALEN-mediated gene editing

Transcription activator-like effector nucleases (TALENs) have a similar basic structure to ZFNs, where the Fok1 nuclease domain is linked to an array of nucleotide-specific DNA binding domains (TAL effectors) originally derived from the plant bacterium *Xanthomonas*. In a similar way to ZFNs, correctly positioned pairs of TALENs promote the formation of an active Fok1 dimer and cleavage of the spacer DNA between the TALEN binding sites. The relatively simple design rules where one TALE unit recognises one nucleotide and the availability of ready-to-clone libraries of DNA recognition units make TALENs a flexible and cost-effective alternative to ZFNs that can be constructed in any molecular biology laboratory (Wright et al. 2014). Since the initial reports demonstrating the utility of TALENs to generate NHEJ (Tesson et al. 2011) and knock-in (HDR) mutant rats (De León et al. 2014), a number of labs have used these gene editors to generate new lines of genetically modified rats for studying cardiovascular biology (Li et al. 2015; Wei et al. 2015; Zhu et al. 2015), neurobiology (Ferguson et al. 2013; Marsan et al. 2016; Tesson et al. 2016) and metabolism (Chen et al. 2016; Yu et al. 2016). These studies and the availability of robust protocols (Tesson et al. 2016) demonstrate that TALENs are an effective, flexible and low-cost platform for directing targeted mutagenesis in the rat.

## CRISPR/Cas gene editing

The clustered regularly interspaced short palindromic repeat (CRISPR/Cas) system is an RNA-dependent adaptive immune system in bacteria and archaea (Horvath and Barrangou 2010; Wiedenheft et al. 2012). In this system, short DNA sequences generated by cleavage of the genome of an infectious agent, such as a phage, are archived within an array (CRISPR) in the bacterial genome. Transcription and processing of the CRISPR array releases CRISPR-RNAs (crRNAs) that direct a nuclease to selectively target and destroy the phage DNA in any future infection. The most commonly used system for gene editing is derived from *Streptococcus pyogenes*, which comprises of a trans-acting crRNA (tracrRNA), a crRNA containing a 20 bp region (protospacer) homologous to the target sequence, and the Cas9 nuclease. The association of the tracrRNA and the crRNA with Cas9 activates the nuclease, thereby promoting crRNA directed cleavage of the target DNA. In an experimental context, the functions of the two RNAs can be combined into a single hybrid gRNA (guide RNA), which means that delivery of a single gRNA and Cas9 protein are sufficient to direct genome editing in any cell type. Site-specific cleavage by the RNA/Cas9 complex also requires that target sequences are abutted by a short sequence recognised by Cas9, the protospacer adjacent motif (PAM). This additional sequence tag, typically 5′-NGG-3′, ensures that in bacteria the system recognises invading PAM-containing DNA, but not host CRISPR-associated protospacer DNA that lacks adjacent PAMs. Since the frequency of 5′-NGG-3′ PAM sequence in the AT-rich rat genome occurs on average every 16–18 bp, much of the rat genome is accessible to CRISPR/Cas-directed gene editing, although in some instances this may prove limiting for knock-in strategies which require the cut site to be less than 20 bp from the intended insertion site (Flister et al. 2015). Furthermore, the short recognition sequence of the CRISPR/Cas system may restrict the targeting of repetitive sequences and closely related genes containing similar sequences.

## CRISPR/Cas-generated knock-out rats

The simplicity and flexibility of the CRISPR/Cas editing system was immediately recognised by groups working with the rat, and is now the preferred editing platform for the standard NHEJ- and HDR-based techniques. Indeed, a major additional advantage of the CRISPR/Cas RNA-based recognition system is that it can be multiplexed. This opens up the possibility of being able to generate multiple mutants in a single round of embryo injections, but has also facilitated the development of more complex targeting strategies.

In 2013, the first reports emerged that described mutant rats generated using the CRISPR/Cas system. Li et al. co-injected gRNAs targeting the gene encoding melanocortin receptors (*Mc3r*/*Mc4r*), and confirmed that the phenotype of a CRISPR/Cas-generated biallelic *Mc4r* mutant rat was similar to a chemically induced *Mc4r* mutant (Li et al. 2013a). In a concurrent report, researchers demonstrated the biological potential of multiplex CRISPR/Cas editing by targeting the Tet gene family of DNA hydroxymethylases (Li et al. 2013b). Injection of combinations of the gRNAs against *Tet1, Tet2* and *Tet3* genes generated rats with compound mutations of all three targets, including Tet1/Tet2 double mutant animals. In contrast, biallelic mutations of the *Tet3* gene were embryonic lethal, demonstrating the essential requirement for Tet3 during embryonic development. This experiment demonstrated the potential of CRISPR/Cas approach to interrogate a biological process such as DNA hydroxymethylation through the simultaneous mutation of multiple genes in the rat. A subsequent report also described similar success in applying multiplex mutagenesis to a more disparate group of genes, *ApoE, B2m, Prf1* and *Prkdc* (Ma et al. 2014b). Co-injection with a mixture of four gRNAs, each targeting one gene, generated 15 pups in which three contained one mutant gene, six contained two mutant genes, five contained three mutant genes and one contained mutations in all four genes.

Initially concerns were raised that the more limited 20 bp gRNA recognition sequence might mean that the CRISPR/Cas system would incur higher levels of off-target cleavage, when compared with the longer 30–36 bp target sequences recognised by ZFN and TALEN dimers. Although systematic comparisons across platforms are not available, the consensus based on reports to date, suggests that the CRISPR/Cas system is no worse than other gene editors. In fact, the limited recognition sequence and its reliance upon accurate hybridisation between gRNA and target DNA means that this system can be used to discriminate between gene variants and selective targeting of specific alleles. In an elegant experiment, Yoshimi et al. turned this to their advantage demonstrating that a single base pair difference between the mutant (albino) and wild-type alleles of the coat colour tyrosinase gene was only targeted by the allele-specific gRNAs (Yoshimi et al. 2014). This was most conclusively demonstrated in vivo, in F1 embryos from albino (F344) × wt (DA) rat crosses, where the injected gRNAs could discriminate between the different alleles. Interestingly, a TALEN designed to target the mutant albino allele could not distinguish between the alleles and cut both equally well. Taken together these experiments showed the sequence specificity of the CRISPR/Cas targeting system and highlighted the potential applications of the system in selectively correcting disease-associated alleles.

## CRISPR/Cas-generated conditional knock-out rats

NHEJ-based mutagenesis using CRISPR/Cas in rat embryos is clearly highly efficient but constitutive mutations can limit analyses when investigating the functions of target genes that are essential for embryonic development or general viability. The ability to conditionally ablate gene function through the Cre/loxP recombination system circumvents this limitation. Using the CRISPR/Cas system to stimulate HDR, researchers have generated conditional alleles following microinjection of Cas9 mRNA/protein and gRNA into zygotes. Ma et al. used circular templates in which the first exons of the DNA methyltransferase enzymes, Dnmt1, Dnmt3a and Dnmt3b were flanked with loxP recombination sites (Ma et al. 2014c). Importantly, in this approach, the loxP sites were positioned so that they disrupted the gRNA target sites, thus protecting the HDR template from cleavage. Interestingly, the use of two gRNAs that cleave at either side of the floxed exon, promoted HDR in approximately 30% of pups targeted at either the *Dnmt3a* or *3b* locus, whereas the efficiency was somewhat lower (16%) for the single cut approach used for the *Dnmt1* gene. Whether the location of the cleavage sites or the use of two gRNAs was important in promoting recombination is not clear from this study. Nonetheless, this group used the same protocol to generate EGFP and CRE-recombinase knock-in rats (Ma et al. 2014a). Using microinjection of circular plasmids as the HDR template along with two gRNAs, the efficiency of correct targeting ranged from 23 to 54% of pups born. Nestin and Cholecystokinin-CRE knock-in rats were crossed with rats carrying floxed *Dnmt* alleles to produce double transgenic F1 rats that had correctly recombined mutant *Dnmt* alleles in the hippocampal region of the brain, thereby demonstrating the utility of these new CRE driver transgenic lines.

In a further elaboration of the Cre/loxp system, Wang et al. inserted an inverted splice acceptor GFP reporter cassette downstream of Exon 1 in the Lgr5 gene by microinjection of Cas9 protein and gRNA mRNA (Wang et al. 2015a). Lgr5 is a marker of stem cell compartments in a variety of tissues including the intestine, stomach and hair follicle (Muñoz et al. 2012) and is a powerful tool in lineage tracing experiments. The GFP reporter cassette, flanked at either side by nested loxP sites (Lox66 and Lox71), was inserted by CRISPR/Cas-directed HDR to generate transgenic founder animals at a frequency of 66%. Subsequent Cre-mediated inversion of the cassette placed the EGFP reporter under the control of the Lgr5 gene resulting in expression of EGFP within the crypts of the rat intestine.

In a recent simplification of the CRISPR/Cas HDR technology, electroporation of long single-stranded DNA (lssDNA) as the HDR template, along with two gRNAs allows efficient generation of conditionally floxed alleles in rat zygotes in the “CLICK” (CRISPR with lssDNA inducing conditional knock-out alleles) method (Miyasaka et al. personal communication). The advantage of the lssDNA template is that it can be quickly and simply prepared from custom-made plasmids using nicking endonucleases. Using a lssDNA to the rat *Vapb* gene (vesicle-associated membrane protein-associated protein B/C), Mashimo and colleagues inserted the P56S mutation associated with amyotrophic lateral sclerosis in humans (Nishimura et al. 2004) into a floxed exon 2 of rat *Vapb*. Half of the rats born carried at least one floxed allele with the P56S mutation. In a further refinement to this methodology, the CLICK method can also be applied to zygotes that carry transgenes driving tissue-specific Cre recombinase, thereby enabling the one-step generation of conditional knock-out animals.

## Large-scale rearrangements using single-stranded oligodeoxynucleotide templates

The previous examples of standard HDR approaches used plasmid DNA templates. However, a number of reports have demonstrated how single-stranded oligodeoxynucleotides (ssODNs) combined with CRISPR/Cas editors can serve as highly selective tools for template-directed mutagenesis (Wang et al. 2013; Yang et al. 2013). To explore the applications of ssODN gene editing in the rat, Yoshimi and colleagues used ssODNs in three different scenarios to manipulate the coat colour genetics of F344 rats: (1) they used a wild-type ssODN to exchange a single base pair in the coat colour gene tyrosinase, thus reverting the albino allele back to wild-type sequence and restoring a non-albino pigmentation pattern, (2) they inserted an additional 19 bp sequence contained in an ssODN into the mutant Agouti-signalling gene *Asipa* locus to recover agouti coat colour and (3) they used two flanking gRNAs to delete a 7000 bp *ERV* retroviral element from the *Kit* gene, using a ssODN containing homology on either side of the junction to bridge across the deletion—thus restoring Kit gene function and a non-hooded pigmentation pattern. The frequency of correctly edited pups in these experiments ranged between 4 and 18% demonstrating the feasibility of using ssODNs to create a variety of precise template-directed mutations in rats (Yoshimi et al. 2014).

**Fig. 2:**
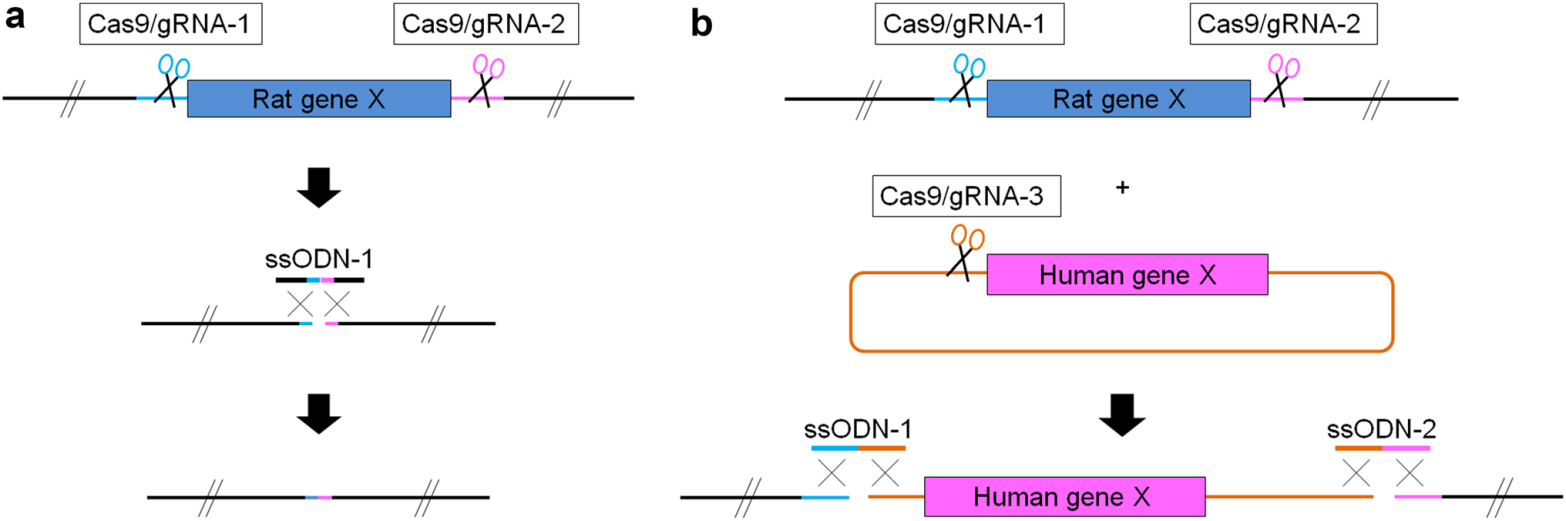
CRISPR/Cas9-mediated large-scale genomic deletions and replacements. Large-scale genomic deletions or replacements can be achieved using pairs of Cas9/gRNAs designed to flank and excise the region to be deleted/replaced. **a** For deletions, a single ssODN containing sequence homology to either side of the cleavage site acts as a ‘bridge’ to paste together the non-adjacent ends. **b** For replacement, a donor plasmid containing the replacement sequence is linearised using a third Cas9/gRNA. Replacement at the desired locus is facilitated by the use of two ssODNs each containing sequence homology to either side of the two cleavage sites bridging the non-adjacent ends

The successful use of an ssODN to paste together two non-adjacent DNA ends, suggested that a similar approach might be effective in joining DNA fragments in trans, for example, ligating an exogenous DNA sequence to the chromosome. This approach in its most simple format requires the cleavage of a genomic target site and a donor plasmid with gRNAs in the presence of two ssODNs that carry the requisite regions of homology to the genome and plasmid sequences (Fig. 2). Using this approach, Yoshimi and colleagues inserted plasmid DNA containing a GFP expression construct into the ROSA26 locus, and placed an entire 200 kb human BAC encoding the anti-phagocytic signal regulatory protein alpha (SIRPA) gene into the rat *Sirpa* locus (Yoshimi et al. 2016). In this latter example, the BAC insertion directed expression of the human gene and eliminated expression of the endogenous rat gene, effectively humanising the rat at this locus. However, retention of the rat gene regulatory sequences may in some instances be an undesirable complication. To cleanly engineer a gene replacement, the endogenous gene can be deleted using two gRNAs, whilst the donor plasmid is linearised with a gRNA, and the appropriate ends of DNA are pasted together using two bridging ssODNs. Using such an approach the entire 58 kb rat *Cyp2d* locus was replaced with the 6.2 kb human *CYP2D6* gene (Yoshimi et al. 2016). A major advantage of using ssODNs to paste together DNA ends is that it eliminates a requirement for lengthy homology arms on the donor DNA. However, a minor limitation of these current schemes is the co-integration of plasmid backbone sequences, along with the donor gene, which may not be desirable since bacterial sequences can attract and seed unwanted epigenetic modifications and have unpredictable consequences on gene expression. Nonetheless, unwanted sequences could be eliminated by gRNA-directed deletion in a second round of gene editing, if necessary.

## Conclusions and future perspectives

The rat has long been recognised as the preferred experimental animal in many areas of biomedical science because of its physiology, behavioural characteristics, size and genetics. Through the recent development of rat ES cell and gene editing technologies, researchers are now in a position to fully exploit the untapped biological potential of this useful laboratory rodent. Using CRISPR/Cas-directed homologous recombination, we can now create specific targeted knock-outs in genes, knock-in transgenes, perform gene replacements and conditionally delete genes at will, opening the way to take full advantage of the wealth of genetic and physiological information that has been accrued since the first studies used rats over a century ago. This will allow us to explore the genetics underlying the biomedically related phenotypes of existing rat strains, and to directly model the genetic basis of many human diseases. Given the rapid pace at which gene editing has developed in the last few years, it is likely that further increases in the efficiency of the CRISPR/Cas system will soon mean that introduction of some types of mutation into the germ line is no longer the limiting factor. However, gene editing increases the efficiency of gene targeting in rat ESCs (Tong et al. 2012; Yamamoto et al. 2015) and therefore CRISPR/Cas-mediated-targeting in ES cells can usefully complement *in ovo* gene editing experiments to enable the pre-selection of biologically relevant mutations and the generation of more complex genetic modifications. ES cells can model many features of early embryonic development and generate many specific cell types in vitro, thus providing the means to interrogate and screen the function of large numbers of candidate genes in biologically relevant cell types. Moreover, the ability to screen many hundreds of clones to identify relatively rare events, and then serially repeat these targeting procedures in culture prior to embarking on experiments in vivo is a significant advantage of using ES cells. For example, these features of ES cells have already been exploited in the implementation of large-scale programmes to completely humanise large sections of up to 6 Mb of chromosomes in the mouse (Wallace et al. 2007; Lee et al. 2014; Macdonald et al. 2014). The application of similar approaches to rat ES cells could be used to humanise significant regions of the rat genome, generating unique models that in a second phase of genetic engineering could be rapidly modified in transgenic rats using standard *in ovo* gene editing techniques. In this way, whilst CRISPR/Cas-mediated gene editing will take the lead in the immediate future, longer-term objectives and more complex projects may benefit from a blend of ES cell and gene editing technologies to deliver the full potential of the rat as a pre-eminent experimental animal model in biomedical research. Incidentally, combining the use of induced pluripotent stem cells together with *in ovo* gene editing will also surely have wider applications in effectively implementing genetic engineering and editing in other commercially important species such as livestock.
